# Supplementary material for: Autonomous robotic searching and assembly of two-dimensional crystals to build van der Waals superlattices
Source: Nat Commun. 2018 Apr 12;9:1413. doi: 10.1038/s41467-018-03723-w (PMC5897399; doi:10.1038/s41467-018-03723-w)
Supplement: Supplementary file 1 — Supplementary Information(PDF 2630 kb) [file 41467_2018_3723_MOESM1_ESM.pdf]

Supplementary Information

Autonomous robotic searching and assembly of two-dimensional crystals to build van der Waals superlattices

Satoru Masubuchi[1,*], Masataka Morimoto[1], Sei Morikawa[1], Momoko Onodera[1], Yuta Asakawa[1], Kenji Watanabe[2], Takashi Taniguchi[2], and Tomoki Machida[1,*]

[1]*Institute of Industrial Science, University of Tokyo, 4-6-1 Komaba, Meguro, Tokyo 153-8505 Japan*

[2]*National Institute for Materials Science, 1-1 Namiki, Tsukuba, Ibaraki 305-0044 Japan*

*Correspondence: mastoru@iis.u-tokyo.ac.jp; tmachida@iis.u-tokyo.ac.jp

Supplementary Figures

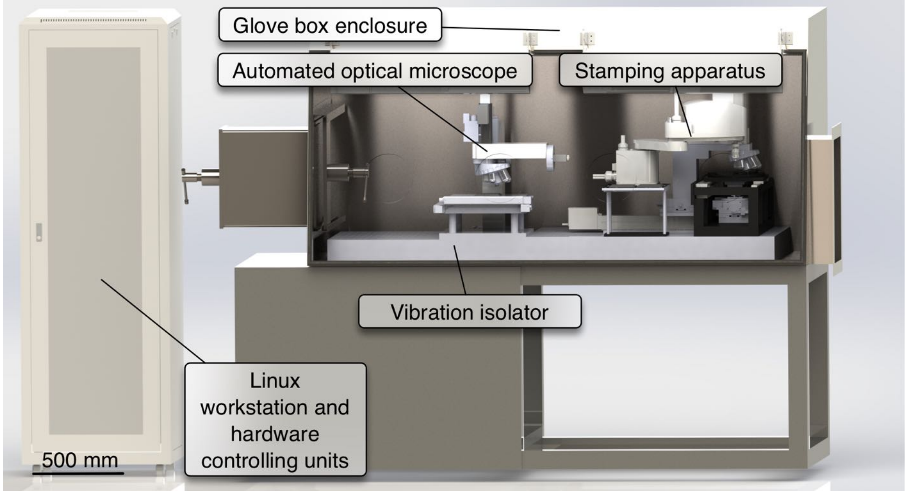

Supplementary Figure 1. **The CAD schematics of our robotic assembly system.**

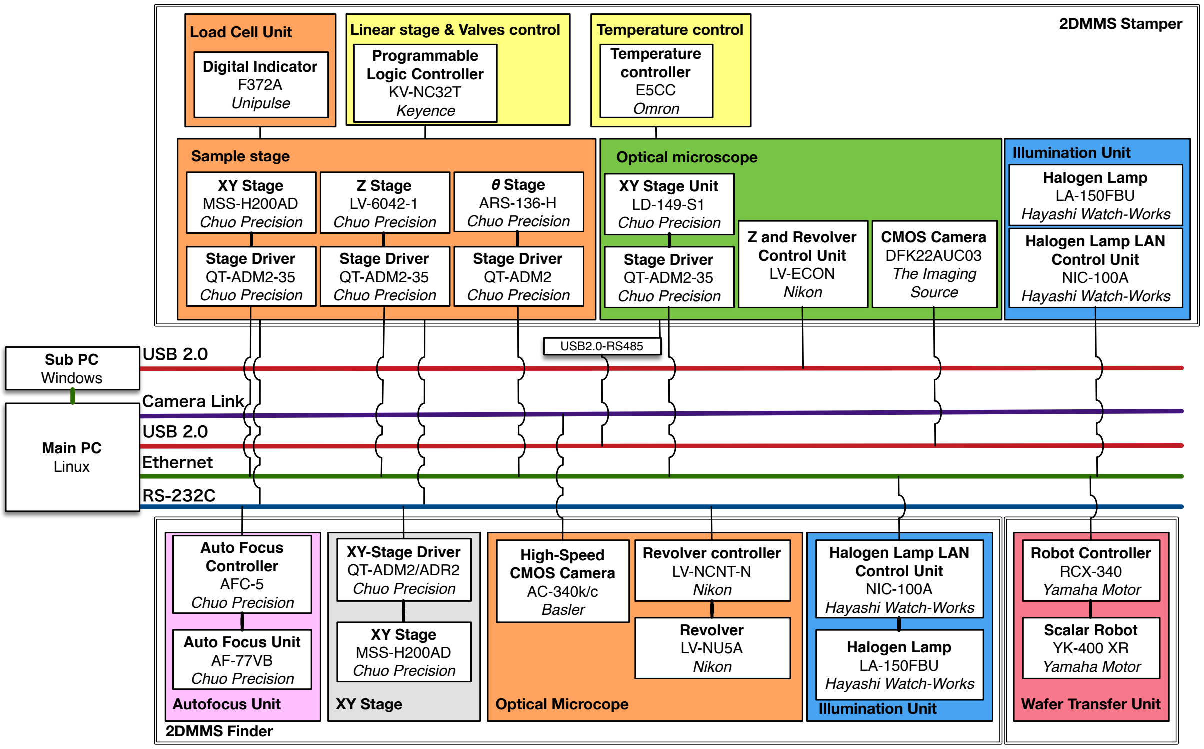

Supplementary Figure 2. **The connectivity diagram of the hardware components utilized in the system.**

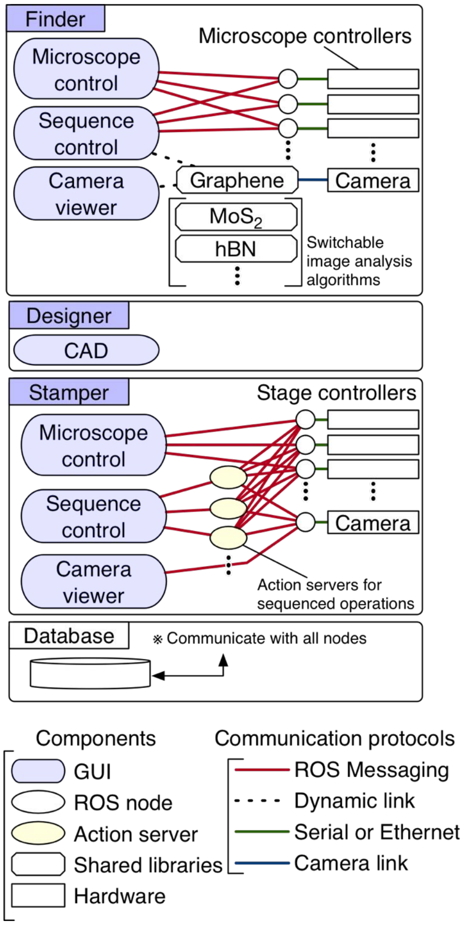

Supplementary Figure 3. **Software connectivity diagram of our automated stamping system.** The GUI consists of three major components: a finder, designer, and stamper. Each GUI sends commands to the ROS action servers and nodes. The image detection algorithms are provided as shared dynamic link libraries; thus, the algorithms can be switched without shutting down the software.

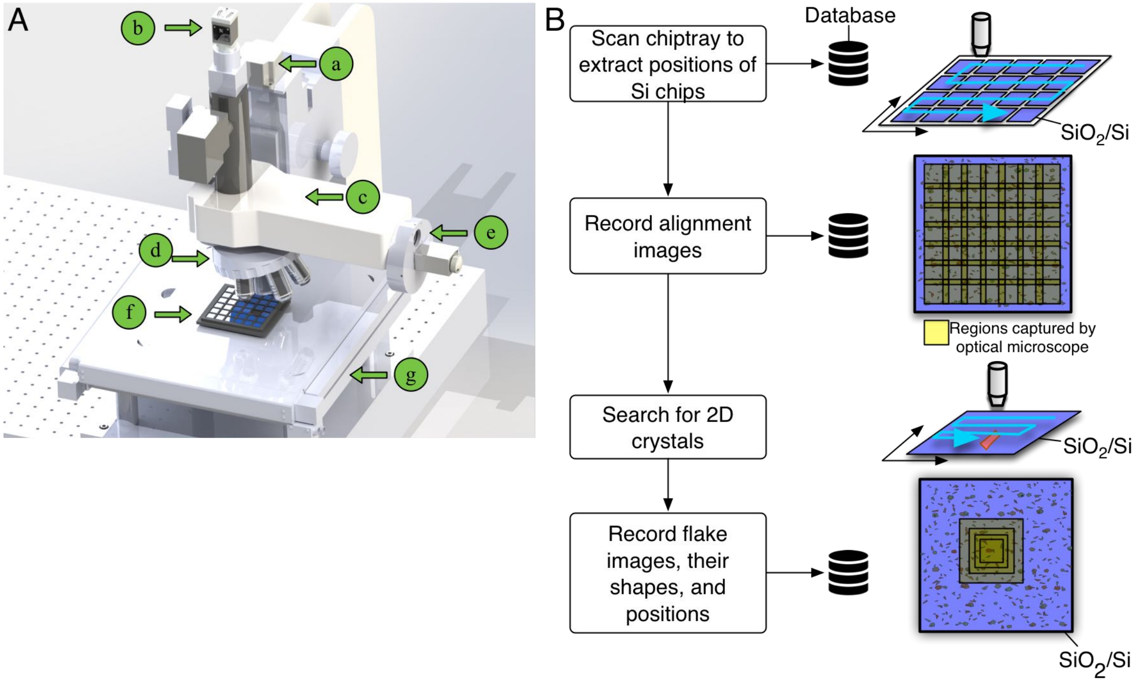

Supplementary Figure 4. **The hardware components and the functionalities of automated searching system (2DMMS-Finder).** (A) Computer-assisted design (CAD) schematics of automated searching system. The searching system consists of the following components: (a) Z-axis scanning stage (Chuo Precision Industrial Co., Ltd.); (b) high-speed CMOS camera (Basler AG, ac2000-340kc); (c) automated focusing unit (Chuo Precision Industrial Co., Ltd., AF-77VB); (d) motorized objective lens (Nikon Corp., LV-NU5A); (e) motorized optical filter wheel (Thorlabs, Inc., FW103H); (f) SiO_2/Si chip tray, which accommodates 36 Si chips (Entegris, Inc.); (g) high-speed motorized XY-scanning stage (Chuo Precision Industrial Co., Ltd., MSS-H200AD); and (not shown) halogen lamp (Hayashi Watch Works Co., Ltd., LA-150FBU). (B) Schematic operational flow diagram of 2DMMS-Finder.

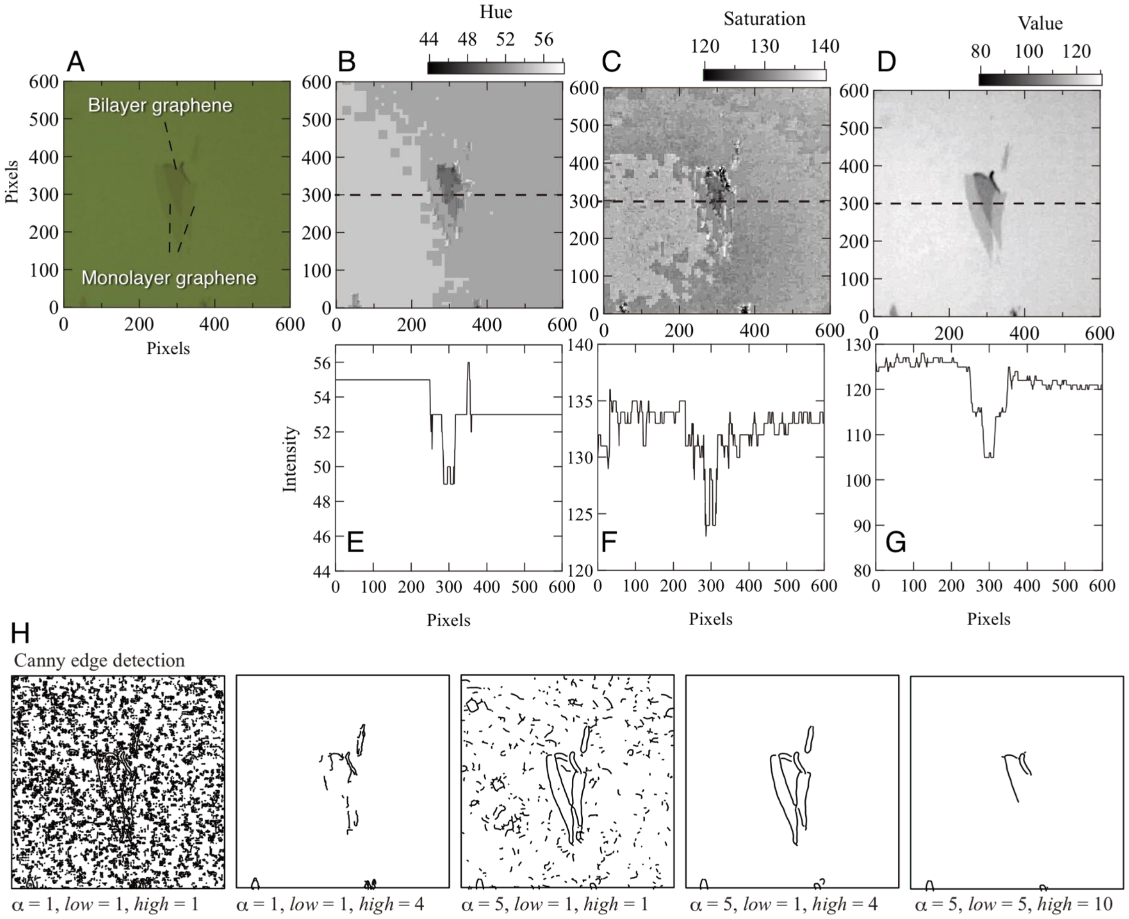

Supplementary Figure 5. **The representative optical microscope image data utilized for tuning the parameters of the flake detection algorithm.** (A) optical microscope image of monolayer and bilayer graphene on SiO_2/Si. (B–D) Intensity mapping of hue, saturation, and value channels, respectively, of the image shown in (A). (E–G) Profile along dotted lines in (B–D), respectively. (H) Results of the Canny edge detection algorithm applied to the image in (A). The edge detection parameters are $(\alpha, low, high) = (1,1,1)$; $(1,1,4)$; $(5,1,1)$; $(5,1,4)$; and $(5,5,10)$.

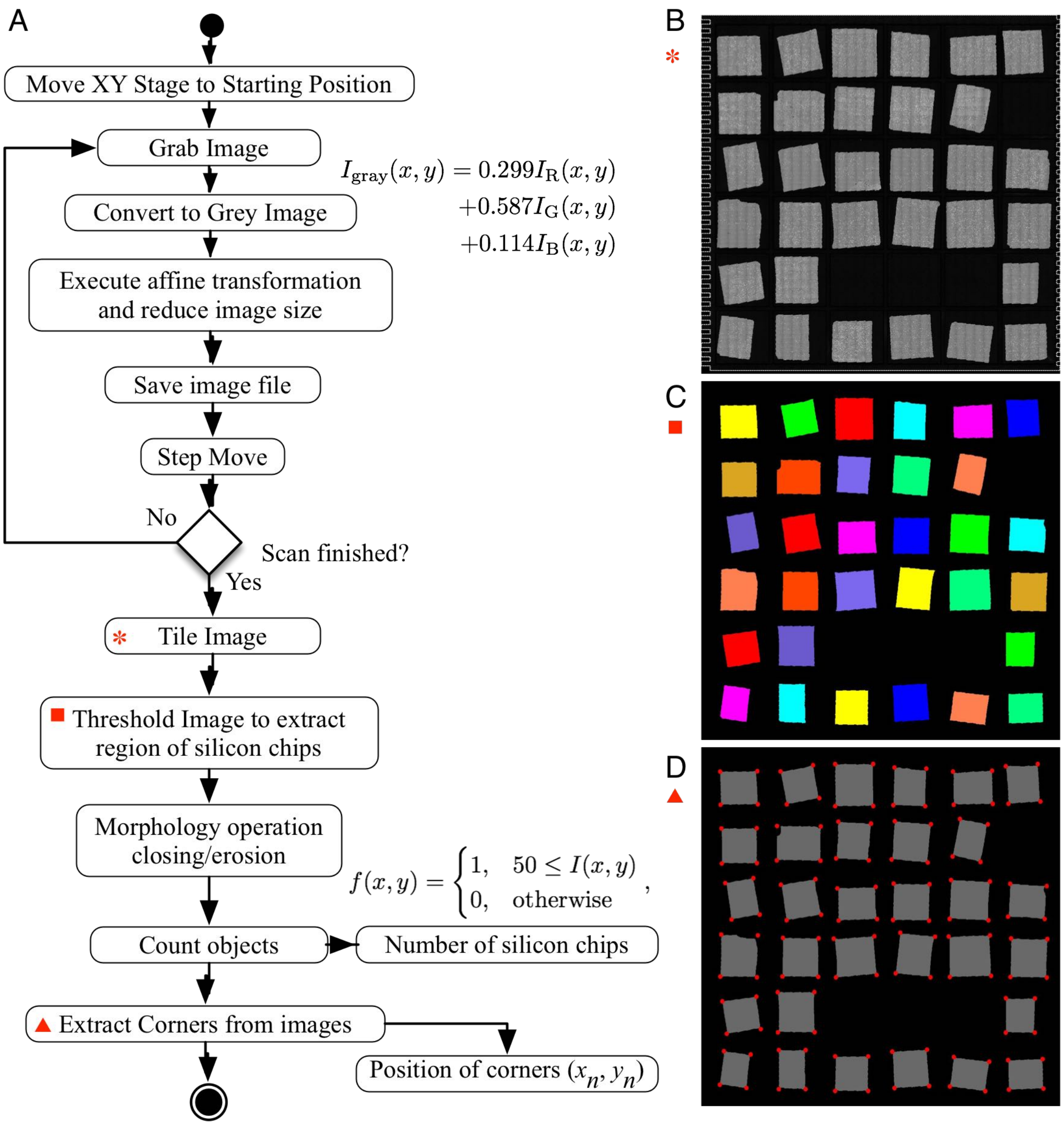

Supplementary Figure 6. **Image processing algorithm used to extract the number and positions of SiO_2/Si chips.** (A) Flow diagram of the algorithm. (B) Tiled grayscale image. (C) Regions extracted as silicon chips. (D) Extracted rectangles that fit the silicon chip regions.

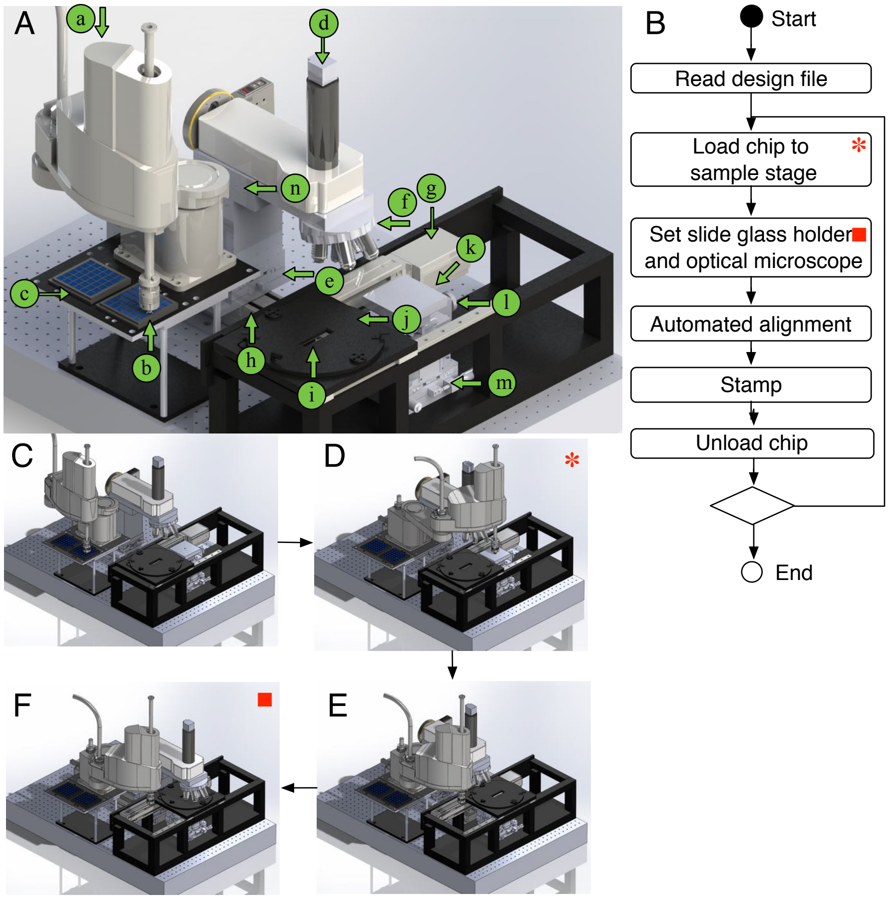

Supplementary Figure 7. **The hardware components and the functionalities of automated assembly system (2DMMS-Stamper).** (A) CAD schematics of our automated stamping system. The hardware components are: (a) a Selective Compliance Assembly Robot Arm (Yamaha Motor Co., Ltd., YK400XR); (b) non-contact wafer tweezers (Solar Research Laboratory Co., Ltd.); (c) SiO_2/Si chip tray (Entegris, Inc.); (d) USB-2.0 CMOS camera (The Imaging Source Asia Co., Ltd., DFK22AUC03); (e) XY-stage unit for optical microscope (Chuo Precision Industrial Co., Ltd., LD-149-S1); (f) motorized objective lens tartlet (Nikon Corp., LV-NU5A); (g) linear sliding stage for optical microscope slide (IAI Corp., ERC2-SA6C-I-PM-6-NP-200-M-NM); (h) linear sliding stage for optical microscope (IAI Corp., ERC2-SA7C-I-PM-4-200-NP-M); (i) slide glass with stamp (homemade); (j) load cell units (TEAC Co., Ltd., TU-PGRS); and (k) sample stage with heater and temperature sensor; (l) θ-stage unit for the sample stage (Chuo Precision Industrial

Co., Ltd., ARS-136-H); (m) XYZ -stage unit for sample stage (Chuo Precision Industrial Co., Ltd., MSS-H200AD and LV-6042-1); (n) Z-stage unit for optical microscope (Nikon Corp., LV-FMA); (o) (not drawn) stabilized halogen lamp unit (Hayashi Watch Works, LA-150FBU); (p) (not drawn) temperature controller (Omron Corp., E5CC); (q) (not drawn) load sensing unit (Unipulse Corp., F372A); (r) (not drawn) programmable logic controller (Keyence Co., Ltd., KV-NC32) to control linear stages, and the solenoid valves for vacuum chucking. Assembly of hardware components d–h and j–r was performed by Nanotech, Inc. Connections to other components and the installation into the glovebox were performed by the author. The controlling programs were developed by the author. (B) Schematic operational steps. (C–F) Step-by step schematic operational steps of the apparatus.

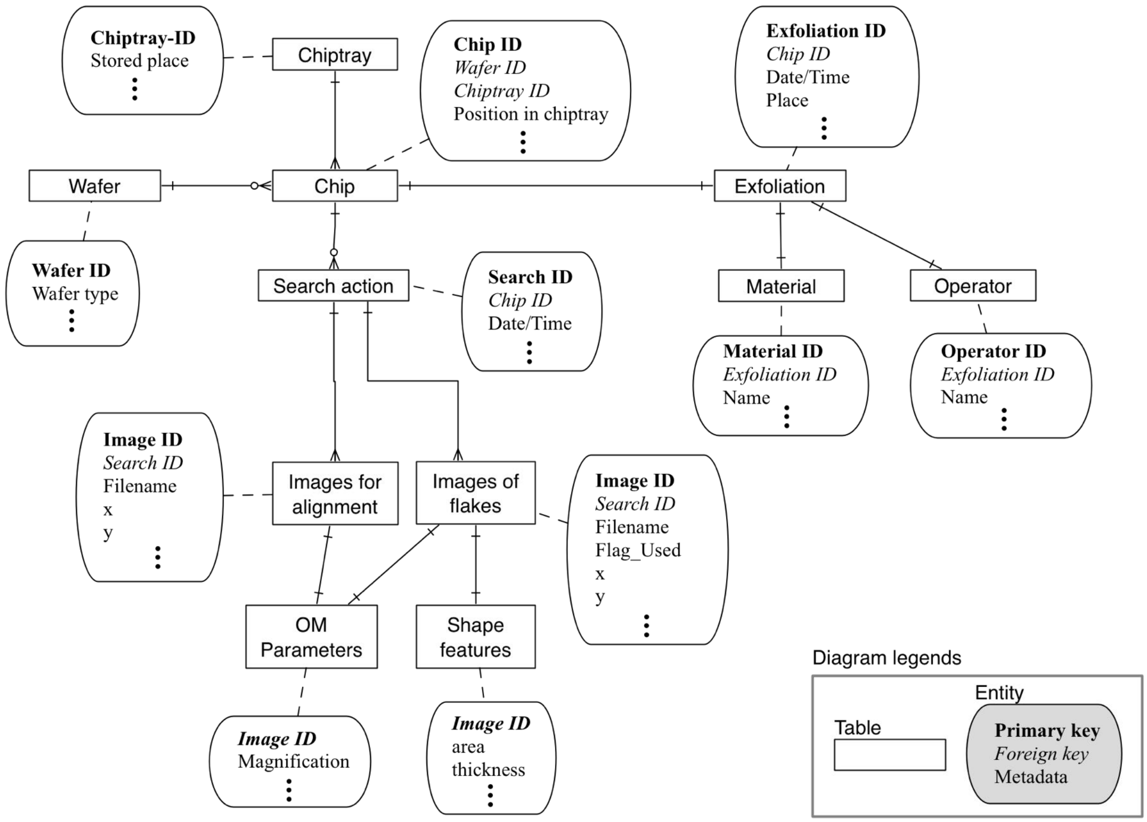

Supplementary Figure 8. **Entity-relation diagram of the database.** The rectangles represent

entities in the database, the connecting lines represent relationships between these entities (1-to-1, 1-to-N, and 1-to-M), and the labels in close proximity to the squares identify the database tables corresponding to the associated entities.

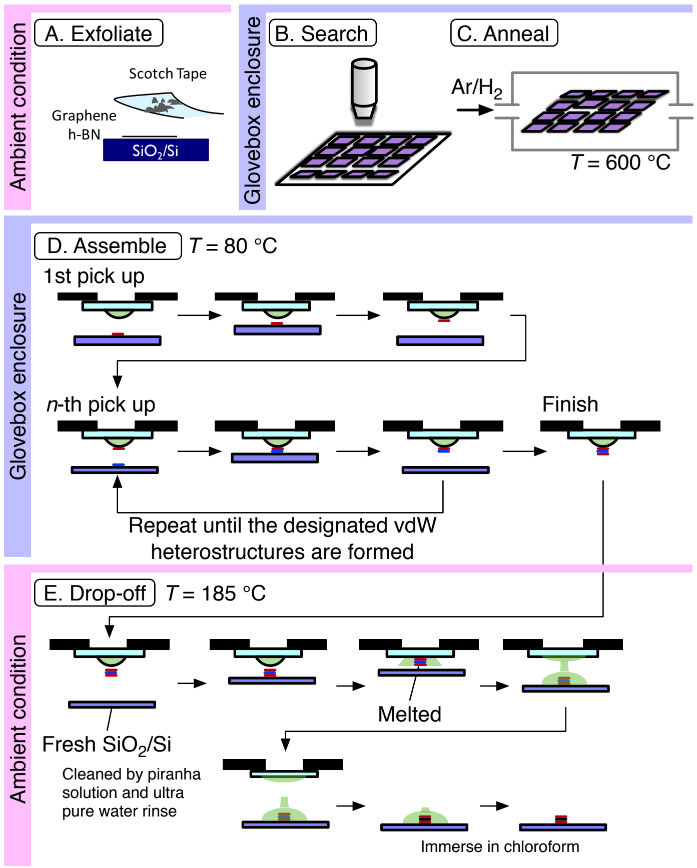

Supplementary Figure 9. **The schematic flow diagram of the fabrication process, including the pre- and post-treatments of the SiO_2/Si substrates.** The process consists of (A) Exfoliation, (B) Searching, (C) Annealing, (D) Assembly, and (E) Drop off. Among them, (A) and (E) were conducted in the ambient air. The processes (B)-(D) were conducted in the glovebox enclosure.

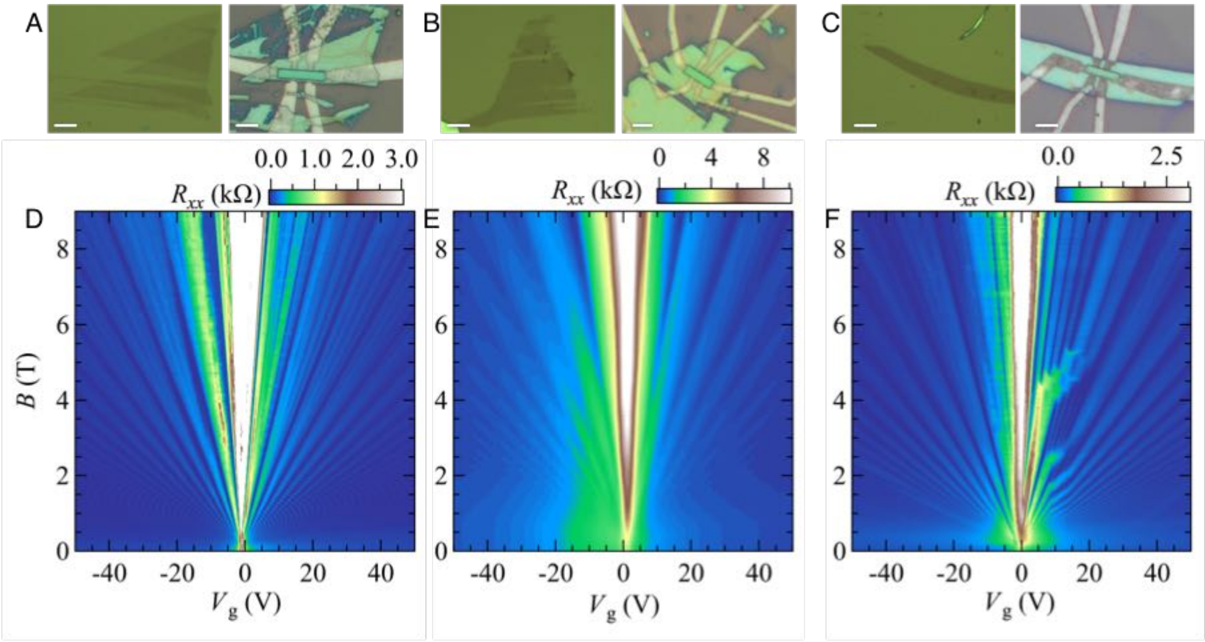

Supplementary Figure 10. **The transport properties utilized for verifying the correspondence between layer thickness and optical color contrast.** (A)-(C) Optical microscope images of (A) monolayer, (B) bilayer, and (C) trilayer graphene devices. The scale bars correspond to 4 μm. (D)-(F) Color plots of the longitudinal resistance R_{xx} measured as a function of back-gate bias voltage V_g and perpendicular magnetic fields B at $T = 1.7$ K for (A) monolayer, (B) bilayer, and (C) trilayer graphene devices.

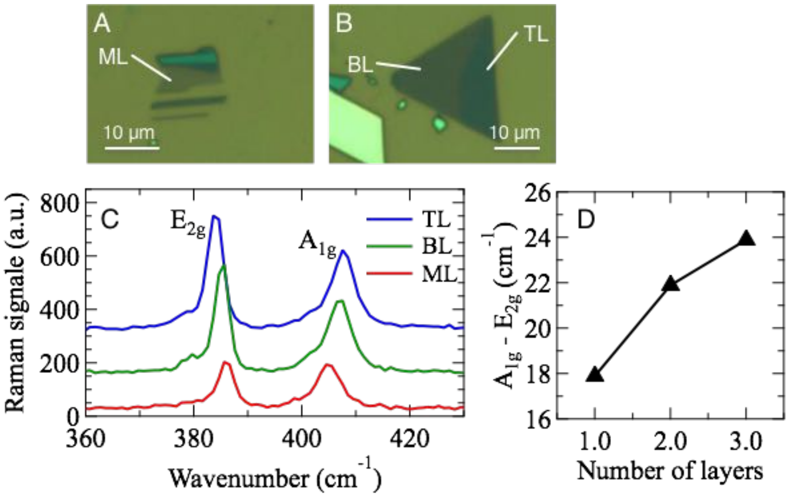

Supplementary Figure 11. **The Raman spectrum utilized for verifying the correspondence between optical color contrast and layer thickness of MoS_2 flakes.** A and B Optical microscope images of monolayer, bilayer, and trilayer MoS_2. Raman spectrum of MoS_2 flakes measured for (A) monolayer, (B) bilayer, and (C) trilayer by using 533-nm laser.

Supplementary tables

**Task**	**time**
**Search graphene and record their positions**	8 hours
**Search hBN and record their positions**	8 hours
**Design heterostructure**	1 hour
**Anneal the sample in Ar/H_2 atmosphere**	4 hours
**Transferring SiO_2/Si chips between experimental apparatus (e.g. transfer silicon chips between chiptray and annealing chamber)**	1 hour
**Assembly**	8 hours
**Miscellaneous tasks such as setting the slide glass, starting up stamping program, operating valves and gas handling systems for annealing apparatus... and so on.**	2 hours
**Total**	**32 hours**

Supplementary Table 1. **The breakdown for the operation time.**

Task	
**Loading silicon chips to the glovebox enclosure**	10 minutes
**Tuning the parameters for detecting graphene and start program**	20 minutes
**Tuning the parameters for detecting hBN and start program**	20 minutes
**Loading silicon chips for annealing chamber**	20 minutes
**Unloading silicon chips from annealing chamber**	20 minutes
**Designing vdW heterostructures**	60 minutes
**Tuning the parameters for assembly and start program**	30 minutes
**Confirm the alignment and perform manual alignment when it's needed**	2x30=60 minutes
**Dropping vdW stack onto the SiO_2/Si substrate**	30 min.
**Miscellaneous tasks; e.g. setting the slide glass, starting up computer, operating valves…**	1.5 hours
**Total**	**6 hours**

Supplementary Table 2. **The task breakdown for the human involvement.**

Condition	Target	C_H	D_H	C_S	D_S	C_V	D_V	Low	High	α	$U_{Entropy}$	L_{Area}
**1**	MLG	0	10	−8	8	−10	4	2	4	1	4.8	300
**2**	BLG	0	10	−14	8	−19	4	2	4	1	4.8	300
**3**	TLG	0	10	−20	8	−28	4	2	4	1	4.8	300
**4**	MLG	0	10	−8	8	−10	4	--	--	--	--	300
**5**	BLG	0	10	−14	8	−19	4	--	--	--	--	300
**6**	TLG	0	10	−20	8	−28	4	--	--	--	--	300

Supplementary Table 3. **Detection parameters utilized to evaluate the performance metrics.**

Condition	n_{total}	n^{det}	$n_{\text{false}}^{\text{det}}$	$n^{\text{det,man}}$	F	τ
1	**56804**	**1203**	**64**	**1131**	**95%**	**4 h**
2	**56804**	**847**	**9**	**---**	**99%**	**4 h**
3	**56804**	**468**	**9**	**---**	**98%**	**4 h**
4	**56804**	**3273**	**1733**	**1131**	**47%**	**4 h**
5	**56804**	**1612**	**357**	**---**	**78%**	**4 h**
6	**56804**	**917**	**45**	**---**	**95%**	**4 h**

Supplementary Table 4. **Performance metrics of 2D crystal detection.** Time elapsed for searching (τ), number of total optical microscope images inspected (n_{total}), number of detections (n^{det}), number of false detections $(n_{\text{false}}^{\text{det}})$, number of flakes detected by inspe manual ction of optical microscope images $(n^{\text{det,man}})$, and percentage of correct answers (F).

Material	# of chips inspected	Elapsed time (h)	Detected	# of false positive (by layer thickness)	# of false positive (by particle)	Percentage of correct answer
Monolayer Graphene	27	4	1886	8	115	93.5
Bilayer Graphene	27	4	1668	8	25	98.0
Trilayer Graphene	27	4	1129	31	7	96.6
Monolayer MoS_2	31	6	362	4	23	92.5
Bilayer MoS_2	31	6	166	4	1	97.0
Trilayer MoS_2	31	6	288	3	1	98.6

Supplementary Table 5. **Spreadsheet summary of the figures utilized for extracting the false detection rate.**

Supplementary notes

Supplementary note 1

Transport characteristics of the fabricated devices

The transport measurements were conducted with a variable-temperature-insert capable of tuning the temperature from $T = 1.5 - 400$ K. Longitudinal resistance (R_{xx}) was measured using the standard lock-in technique with an alternating current of $I_{\mathrm{ac}} = 10$ nA. An Si substrate was utilized as a global back-gate to tune the charge carrier density, $n_e = C_{\mathrm{g}}(V_{\mathrm{g}} - V_{\mathrm{Dirac}})$, where C_{g} is the gate capacitance, $C_{\mathrm{g}} = 9 \times 10^{-8}\ \mathrm{Farad/cm^2}$; V_{g} is the gate bias voltage; and V_{Dirac} is the value of V_{g} at the charge neutrality point.

We present the transport characteristics of the following three devices fabricated by 2DMMS: [Supplementary Figure 12A] monolayer graphene encapsulated in hBN, [Supplementary Figure 12B] monolayer graphene with crystallographic alignment to hBN crystals, and [Supplementary Figure 12C] graphene/bilayer hBN/bilayer graphene with vertical tunneling. In Supplementary Figure 12A, the R_{xx} vs. V_{g} curve shows a narrow peak with a full width at half maximum of $\delta V_{\mathrm{g}} \approx 0.2$ V, indicating a small inhomogeneity in the charge carrier density. The charge carrier mobility reached $\mu \approx 1{,}000{,}000\ \mathrm{cm^2/Vs}$, which is comparable to those reported in the highest quality devices[18]. Supplementary Figure 12B shows R_{xx} versus V_{g} for the aligned monolayer graphene/hBN heterostructures. The presence of peak structures at $V_{\mathrm{g}} = \pm 40$ V indicates second-generation charge neutrality points due to a Moiré superlattice, demonstrating that the crystallographic orientation between graphene and hBN flakes can be successfully tuned by our system. Supplementary Figure 12C shows the differential conductance dI/dV_{sd} measured between graphene and bilayer graphene through bilayer hBN as a function of V_{g} and the source-

drain bias voltage V_{sd}. A region of suppressed conductance is observed at approximately $V_{sd} = 0$ mV, indicating the realization of tunneling transport through thin hBN.

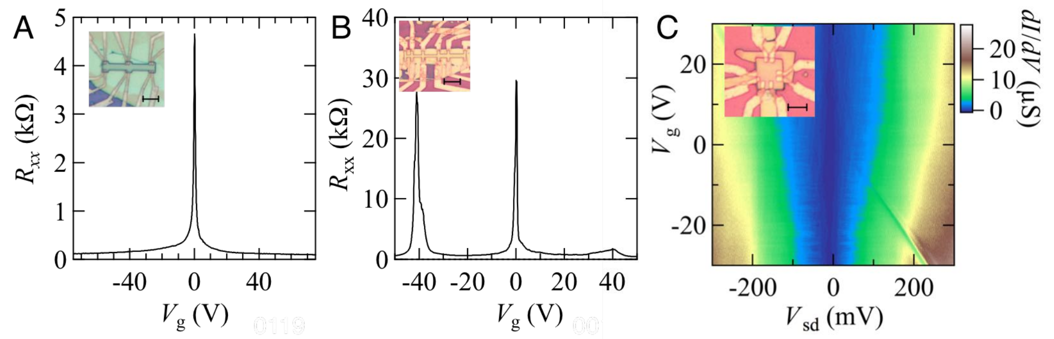

Supplementary Figure 12. **Evaluation of transport characteristics of the fabricated devices.** (A) Longitudinal resistance (R_{xx}) of monolayer graphene encapsulated in hBN as a function of back-gate bias voltage (V_g) measured at T = 4.2 K. (B) R_{xx} as a function of V_g for aligned graphene/hBN heterostructures measured at $T = 4.2$ K. (C) Color plot of differential conductance dI/dV of the graphene/bilayer hBN/bilayer graphene tunneling device as a function of V_g and bias voltage applied between the monolayer and bilayer graphene V_{sd}. The blue region indicates suppressed conductance. (A–C) Insets indicate the optical microscope images of the measured devices. The scale bars indicate 2 μm.

Supplementary note 2

Evaluation of the charge carrier mobility in BN/G/BN heterostructures

We performed transport measurements in two hBN/graphene/hBN heterostructures at $T = 4.2\ K$, which we call the device I and II, respectively. The optical microscope images of devices I and II were shown in Supplementary Figure 13 A and E, respectively. In both devices, the longitudinal resistance (R_{xx}) as a function of back-gate bias voltage (V_g) exhibited sharp peak structure with small full width at half maximum of $\delta V_g = 0.25$ V and 0.5 V, indicating the small

charge carrier inhomogeneity [Supplementary Figure 13 B and F]. The R_{xx} was converted to the longitudinal resistivity using $\rho_{xx} = \frac{W}{L} R_{xx}$, where W and L are the channel width and length. The longitudinal conductivity $\sigma_{xx} = 1/\rho_{xx}$ as a function of V_{g} were shown in Supplementary Figure 13 C and G. From σ_{xx}, we calculated the charge carrier mobility using the Drude model $\mu = \sigma/ne$, where n is the charge carrier density. The value of n was calculated by using $n = C_g(V_g - V_{\mathrm{Dirac}})$, where $C_g = 1 \times 10^{-4}\ \mathrm{F/m^2}$ is the gate capacitance, and V_{Dirac} is the position of charge neutrality point. In the entire range of V_{g}, the values of charge carrier mobility exceeded $\mu > 10^6\ \mathrm{cm^2/Vs}$, indicating the high quality of our devices.

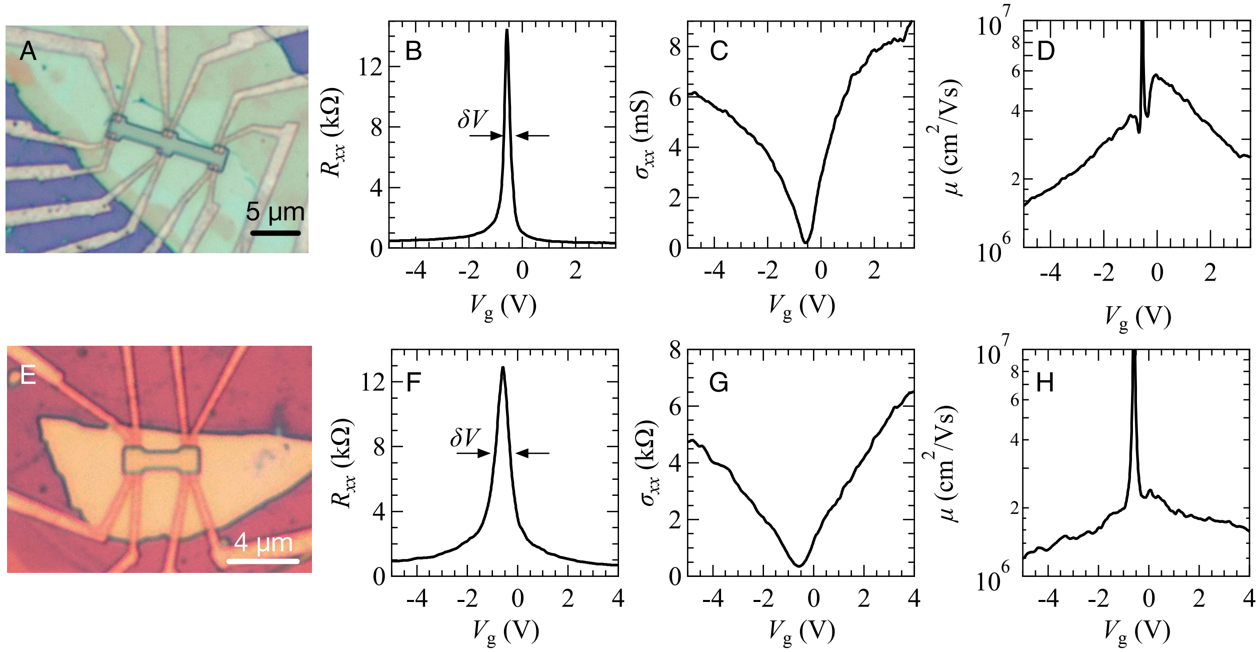

Supplementary Figure 13. **Evaluation of the charge carrier mobility of BN/G/BN heterostructures.** (A and E) Optical microscope images of monolayer graphene encapsulated in hBN. (B and F) Longitudinal resistance (R_{xx}) as a function of back-gate bias voltage (V_{g}) measured at $T = 4.2$ K. (C and G) Longitudinal conductivity (σ_{xx}) as a function of V_{g}. (D and H)

Charge carrier mobility μ as a function of V_g. The data in A-D (E-H) were obtained in the device I (II).

Supplementary note 3

Evaluation of the interface cleanness of the fabricated vdW heterostructure

In order to evaluate the interface cleanness of the fabricated vdW heterostructures, we assembled the hBN, WS_2, MoS_2, and hBN flakes [Supplementary Figure 14A] to hBN/WS_2/MoS_2/hBN heterostructure [Supplementary Figure 14 B], and conducted the photoluminescence measurements. Supplementary Figure 14 C shows the AFM image in the region surrounded by the black dashed square in Supplementary Figure 14 B. The photoluminescence spectra were taken by using a 533-nm laser to excite the monolayers of MoS_2, WS_2 and MoS_2/WS_2 heterostructure. The laser beam was focused to a spot size of $\sim$ 1 μm with a total power of 1 mW. A monochrometer and a Peltier-cooled charge-coupled device (CCD) were used to record the photoluminescence spectra. The photoluminescence spectra measured at monolayers of MoS_2 (green) and WS_2 (blue curve) were shown in Supplementary Figure 14 D. They exhibited narrow peak structure with half width at half maximum of $\Delta\lambda = 14$ nm for monolayer MoS_2 and $\Delta\lambda = 6.6$ nm for monolayer WS_2, which is comparable to the best quality device reported to date [F. Cadiz et al., PRX **7**, 021026 (2017)]. In addition, in the spectrum taken at MoS_2/WS_2 heterostructure (red curve), the photoluminescence from the monolayer MoS_2 and WS_2 bands $\lambda = 664$ and 624 nm, respecteively, were significantly suppressed. This observation demonstrates the coupling between the MoS_2 and WS_2 [Y. Gong *et al.*, Nature Materials **13**, 1135 (2014)].

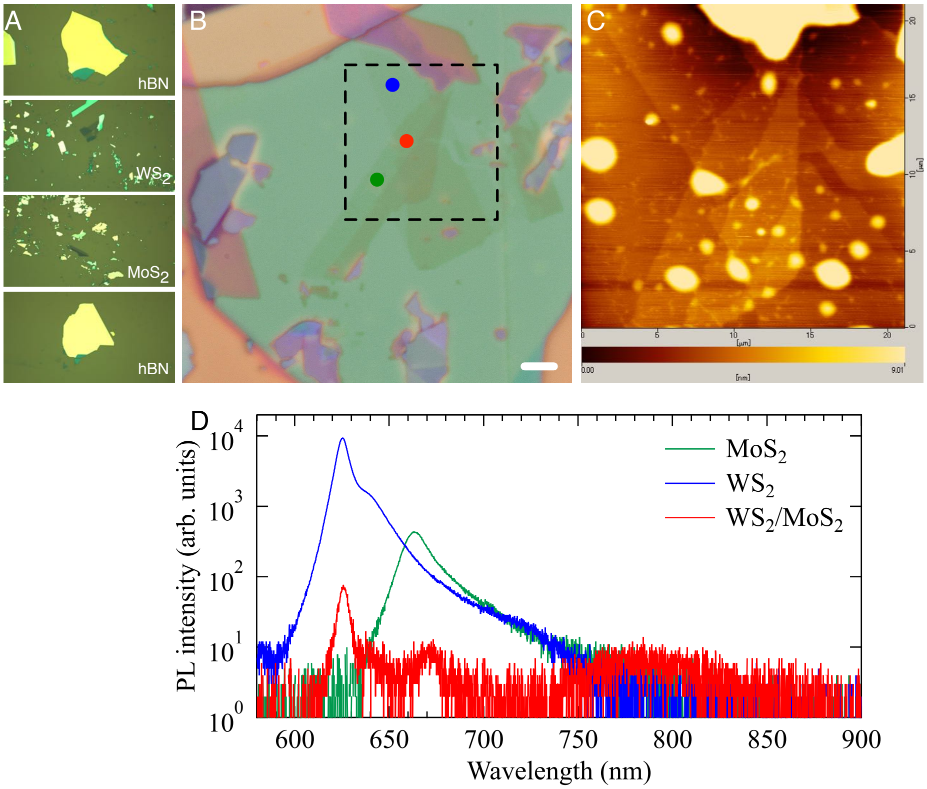

Supplementary Figure 14. **Evaluation of the optical properties of MoS_2/WS_2 heterostructure.** (A) Optical microscope images of the hBN, WS_2, MoS_2, and hBN flakes (top to bottom) utilized for assembling WS_2/MoS_2 heterostructures. (B) Optical microscope image of the hBN/WS_2/MoS_2/hBN heterostructure. The scale bar corresponds to 5 μm. (C) Atomic force microscope image of the region surrounded by the black dashed square in B. (D) Photoluminescence spectrum measured in the regions of WS_2 (blue), MoS_2 (green), and WS_2/MoS_2 (red). The correspondence to the measurement points were indicated by the colored circles in B.

Supplementary note 4

Formation of the bubbles in the fabricated vdW heterostructures

We fabricated the three vdW heterostructures; hBN/4L-graphene/hBN [Supplementary Figure 15 A], hBN/graphene/4-5L hBN/trilayer graphene/hBN [Supplementary Figure 15 B], and

hBN/MoS_2/WS_2/hBN [Supplementary Figure 15 C]. The AFM images of the regions surrounded by the black dashed rectangles in Supplementary Figure 15 A-C were presented in Supplementary Figure 15 D-E. In hBN/4L-graphene/hBN [Supplementary Figure 15 A], the channel region was free of bubbles [white dashed rectangle in Supplementary Figure 15 D]. In hBN/graphene/4-5L hBN/trilayer graphene/hBN [Supplementary Figure 15 B], some bubbles were discerned [Supplementary Figure 15 E], however sufficiently large area $> 2 \times 2$ μm was still remained intact, and can be provided for the tunneling transport studies. In the case of hBN/MoS_2/WS_2/hBN [Supplementary Figure 15 C], the situation is similar to the hBN/graphene/4-5L hBN/trilayer graphene/hBN. There were some bubbles, but the sufficiently large area was remained intact [Supplementary Figure 15 F].

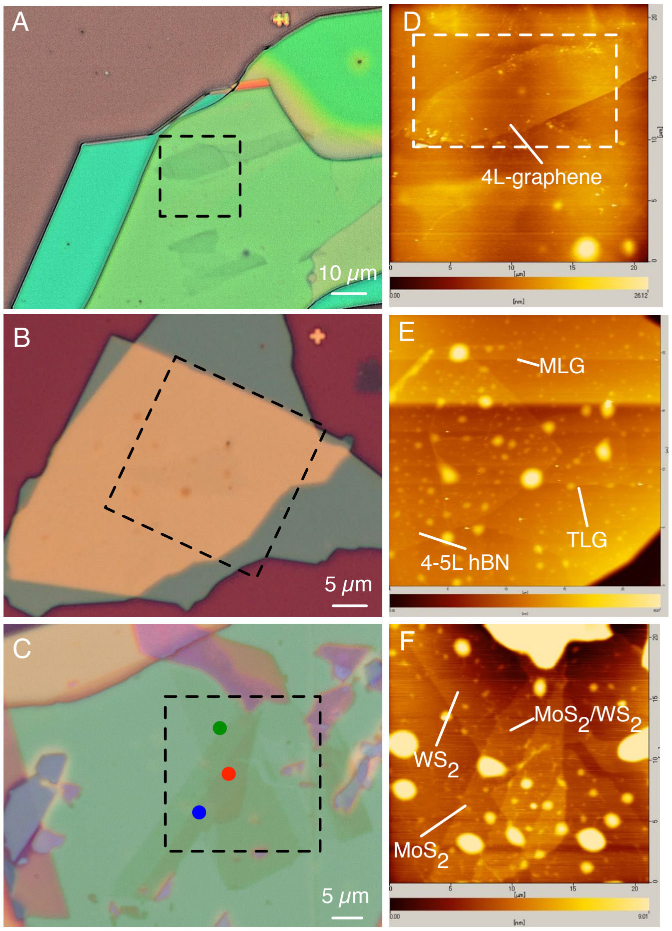

Supplementary Figure 15. **Evaluation of bubbles.** (A-C) Optical microscope images of (A) the tetra layer graphene encapsulated in hBN, (B) hBN/graphene/4-5L hBN/trilayer graphene/hBN, and (C) hBN/MoS_2/WS_2/hBN. (D-F) AFM images of the regions surrounded by the black dashed rectangles in (A-C).

Supplementary note 5

Effects of entropy thresholding on the 2DMMS-Finder performance metrics

We conducted automated searching for monolayer, bilayer, and trilayer graphene on 18 SiO_2/Si chips using the detection parameters summarized in Supplementary Table 3. The parameters were divided into two groups: with and without entropy thresholding; conditions 1–3 and 4–6, respectively. The resulting performance metrics are summarized in Supplementary Table 4. In

Supplementary Table 4, the detection with the entropy thresholding is shown to result in a high percentage of correct answers, > 95%. In contrast, the detection without entropy thresholding results in a significantly decreased percentage of correct answers, especially for the case of monolayer graphene (47%). This result was caused by the false detections produced by the vertices of shadows generated by contaminating objects, as presented in Supplementary Figure 16.

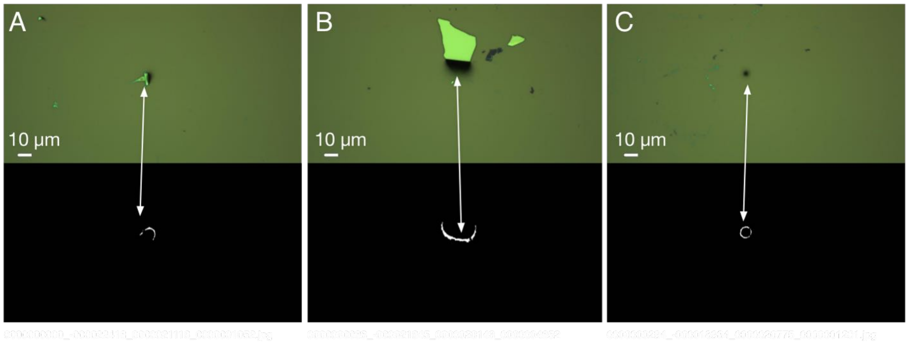

Supplementary Figure 16. **Examples of false detection** (A–C) The false detection generated by condition 4 in Supplementary Table 3 エラー! 参照元が見つかりません。. The top panels show input optical microscope images; the bottom panels show regions extracted as 2D crystals. The shadows generated by contaminating objects often result in false detections.

Supplementary note 6

Remarks on the difference between the entropy and color thresholding.

To explain the difference between the images F and G, in エラー! 参照元が見つかりません。, we summarized the response to the monolayer graphene, the bilayer graphene, corrugated crystals, and the tape residues in Supplementary Table 6. In general, the entropy thresholding gives positive response to the flat objects, which is surrounded by the edges. Therefore, it gives positive response to the monolayer graphene, bilayer graphene, and tape residues [Supplementary Table 6 and Figure 3F]. On the other hand, the color thresholding extracts the region where the optical

intensity is within the set range. Therefore, it shows positive response to monolayer graphene and corrugated crystals [Supplementary Table 6 and Figure 3G]. Although the difference between extracted images were apparently slight, however by taking the intersection between the images F and G, only the region of monolayer graphene was extracted, thereby demonstrating the different nature in these thresholding algorithms. The robustness of the detection algorithm to the contaminating objects has been provided by this mechanism.

	Entropy thresholding [Fig. 3 F]	Color thresholding [Fig. 3G]	The intersection between F and G
**Monolayer graphene**	Positive	Positive	Positive
**Bilayer graphene**	Positive	Negative	Negative
**Corrugated crystals**	Negative	Positive	Negative
**Tape residues**	Positive/Negative	Negative	Negative

Supplementary Table 6. The typical response of the entropy and color thresholding to the monolayer graphene, bilayer graphene, corrugated crystals, and tape residues.

Supplementary note 7

The examples of failed transfer.

In the main text, we commented on the high success rate for the transfer. We consider thatit is fair to show some examples of the cases which fail. The most common result when we fail is that the targeted 2D crystals are remained on the surface of SiO_2/Si substrate after contacting the host 2D crystal to the targeted 2D crystal, as schematically illustrated in Supplementary Figure 17. To illustrate some example of the failure, we show the several targeted 2D crystals that could not be picked up in Supplementary Figure 18A-C. In all cases, the host crystals were the single crystals of hBN. In the case of Supplementary Figure 18A, the thick region of hBN [white arrow in Supplementary Figure 18A] was picked-up whereas the thin hBN [red arrow in Supplementary

Figure 18A] was remained on the substrate. In the case of Supplementary Figure 18B, the similar failure took place; the thick region [white arrow in Supplementary Figure 18B] was lifted but the other area was remained on the substrate. As a reason of these failures, we speculate that the thick 2D crystal prevented targeted 2D crystal to be contacted to the host 2D crystals, thereby remaining it on the SiO_2/Si substrate. In the case of Supplementary Figure 18C, the whole structure of trilayer graphene [red arrow in Supplementary Figure 18C] was not lifted. In this case, the targeted flake was kept in the ambient air for more than 1 month before annealed in the Ar/H_2 gas. Therefore, the surface of targeted 2D crystals were presumably be contaminated.

Nevertheless, if we load the 2D crystals immediately after the exfoliation, perform annealing inside the glove box, and select the 2D crystals so that they were well separated from the other 2D crystals, we achieved almost perfect success rate. In the previous setup built in the ambient air, the success rate was gradually decreased, and after the several hours of the exposure of the targeted 2D crystal to the ambient air, the success rate was decreased to almost zero, which is presumably due to the contamination accumulated on the surface of the targeted 2D crystal during the fabrication process.

We speculate that the reason of high success rate realized in this system owes to the fact that the most time-consuming part of the fabrication process (searching and transfer) were conducted entirely inside the glove box enclosure, which prevents contaminating the surface.

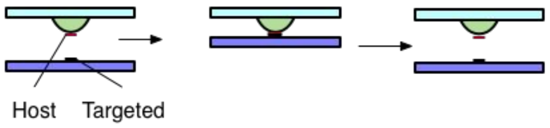

Supplementary Figure 17. The schematic illustration of the typical cases which we fail.

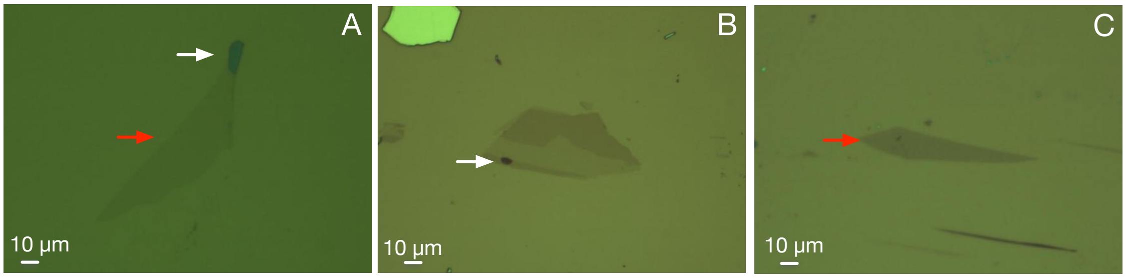

Supplementary Figure 18. Representative optical microscope images of the 2D flakes that we failed to pick-up. The materials were (A) thin hBN, (B) hybrid monolayer and trilayer grpahene, and (C) trilayer graphene. The trilayer graphene (C) was exposed to the ambient air for more than a month before annealing in Ar/H_2 gas.

Supplementary note 8

Remarks on the final alignment process.

When we performed the automated alignment process, it can bring the targeted 2D crystals within a lateral error of 10 µm, and if this order of tolerance can be afforded, the system can stack the 2D crystals in a fully automated way. The stamping demonstration presented in supplementary movie S4 was conducted by just clicking the confirmation button at 00:53 and no manual alignment was performed. In addition, we intentionally implemented the system so that the human operators can override the alignment process even during the automated lifting process of SiO_2/Si chip. We believe that this strategy is favorable for good balancing between the flexibility and concreteness, and for the device production in the fundamental scientific research activity. We consider that the fully automated alignment is an optional functionality, which will be implemented in the future version of the software system.

Supplementary note 9

Selection of the straight edges on the two-dimensional crystals

In the CAD software, the crystal orientation was inferred relying on the straight edges of

the 2D crystals. The selection of the flakes was performed by an operator. To illustrate the fitting process, we present the screenshots of the CAD software [Supplementary Figure 19]. Supplementary Figure 19A shows the optical microscope image of the exfoliated hBN flake. The edge patterns extracted from Supplementary Figure 19A are presented in Supplementary Figure 19B. The operator draws a box on the computer screen [white arrow in Supplementary Figure 19B]. Then the straight line is fitted to the edge pattern by using the least-squares method [Supplementary Figure 19C]. The extracted angle of the straight line to the horizontal axis was (i) $\theta = 17.4215°$. The other edges can also be fitted as (ii) $\theta = 77.2986°$, and (iii) $\theta = 17.5254°$ [Supplementary Figure 19D]. The relative angle between (i) and (ii) was $\Delta\theta_{\mathrm{i-ii}} \sim 59.87°$, whereas that between (i) and (iii) was $\Delta\theta_{\mathrm{i-iii}} = 0.1°$. Note that, these values reflect the crystallographic symmetry of hBN, where the expected exfoliation angles were $\Delta\theta_{\mathrm{i-ii}} = 60°$ and $\Delta\theta_{\mathrm{i-iii}} = 0°$. From these results, we estimate the extent of error in the fitting process to be $\sim 0.1°$.

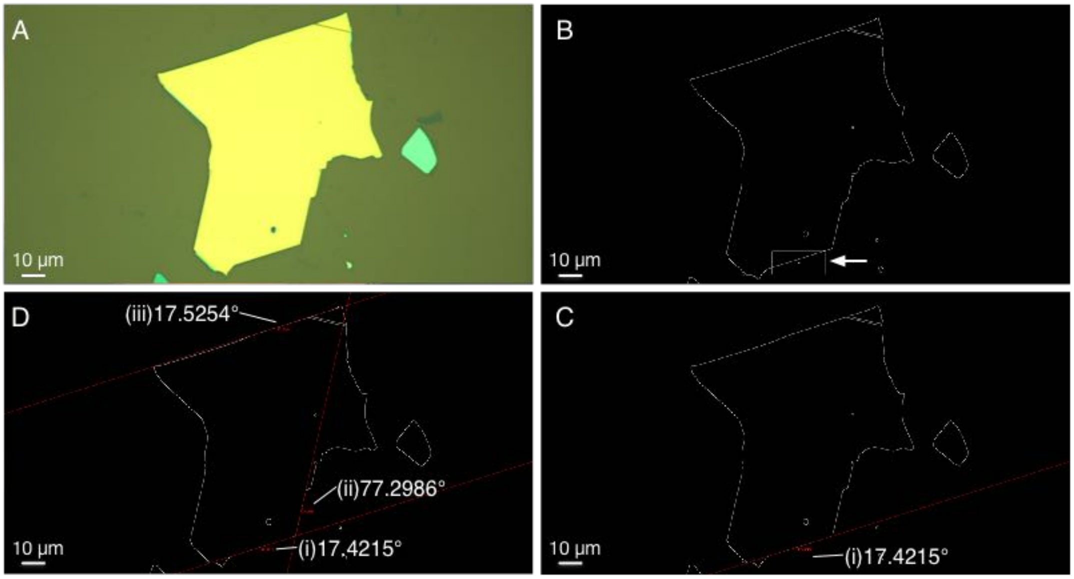

Supplementary Figure 19. The demonstrations of the edge fitting. The presented images are the screenshots of the software. (A) The representative thick hBN flake. (B) (white curves) the edge

patterns extracted from the image A. The rectangle highlighted by the white arrow indicates the region that are going to be fitted by the straight line. (C) The extracted angle between the horizontal line was 17.4215°. (D) The extracted angles of the other edges relative to the horizontal line were 77.2986° and 17.5254°.

Supplementary note 10

Accuracy of the alignment in terms of the rotational shift

To illustrate the accuracy and reproducibility in the rotational angle between flakes, we provide in Supplementary Figure 20, the comparison between the designed (A and B) and obtained angles (C and D) for the two representative devices numbered 0110 and 0112. In the device 0110, the alignment angles between the set of straight edges were designed as 3.0° [Supplementary Figure 20 A]. The measured angles between the corresponding straight edges in the fabricated device were 2.57° [Supplementary Figure 20 C]. In the device 0112, the alignment angles between the set of edges were designed as 40.3° [Supplementary Figure 20 B] and the result of measurement was 39.0° [Supplementary Figure 20 D]. However, note that the fitting to the straight edges in the optical microscope images were conducted by hand-fitting. From these data, the accuracy of the alignment angle can be estimated to be less than 1.5°

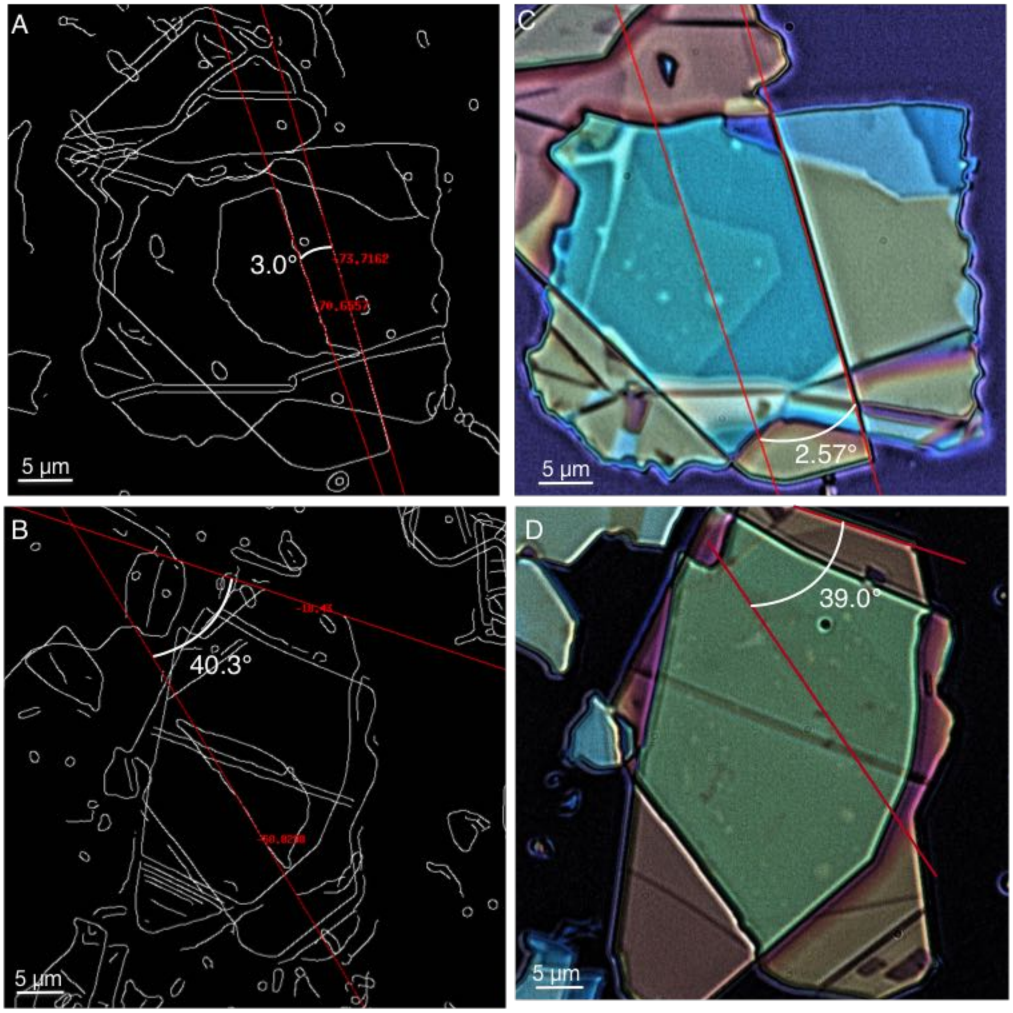

Supplementary Figure 20. A and B, the relative angles between the straight edges in the vdW heterostructures design. C and D the optical microscope images of the fabricated vdW heterostructures. Note that, in these devices, translational positions were intentionally shifted with respect to the designed position during the assembly. Therefore, the alignment error in the translational direction needs be ignored.

Supplementary note 11

Accuracy of the alignment in terms of the translational shift

To demonstrate the of alignment in terms of the translational shift, we conducted the alignment

demonstration as presented in Supplementary Figure 21. Supplementary Figure 21A-H shows the image of the hBN crystal in the CAD software screen. In these designs, the alignment center was locked to the fixed position on the hBN flake, as indicated by the red lines in Supplementary Figure 21A-H. To demonstrate the alignment accuracy, each design was rotated by the step of $\theta = 45°$. The result of automated alignment in the stamping apparatus were presented in Supplementary Figure 21I-P. Note that, during the alignment, no human intervention was made. The targeted flake was aligned to the designated position within a lateral error of 10 μm.

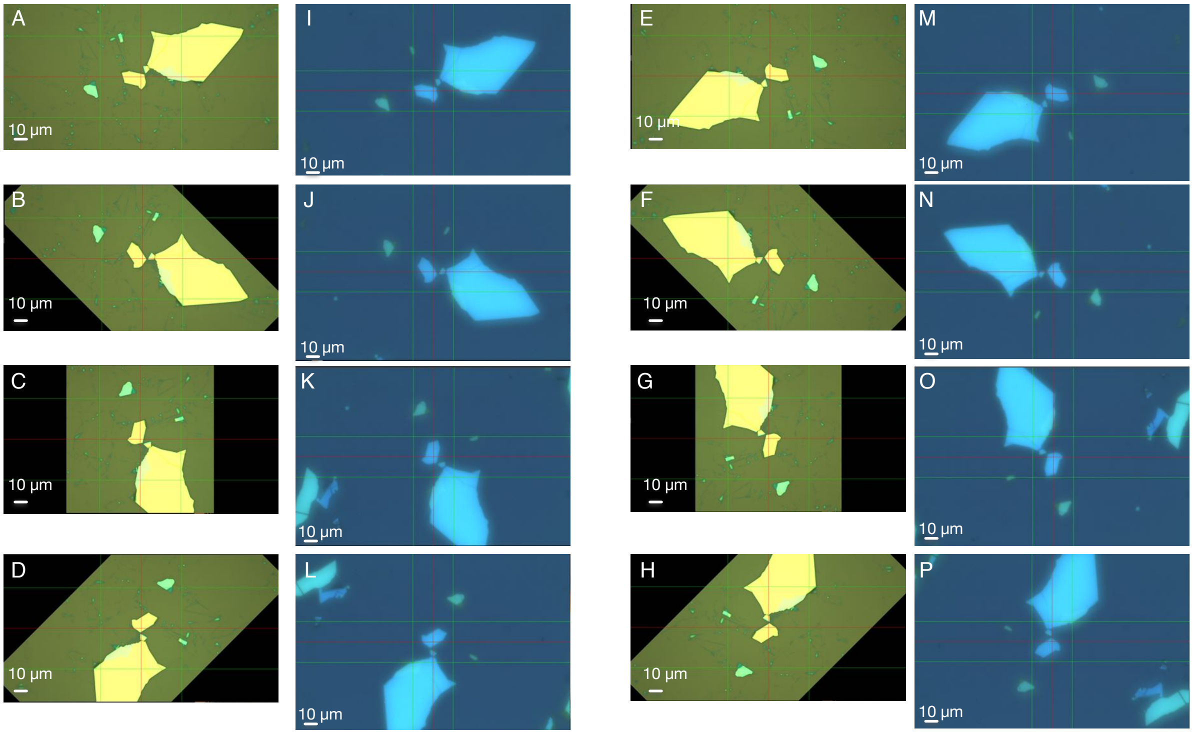

Supplementary Figure 21. The demonstration of the alignment. (A-H) The optical microscope image of the hBN crystal in the CAD screen. The hBN flake was rotated by a step of $\theta = 45°$. The targeted alignment center was indicated by the red straight lines. (I-P) The optical microscope image after alignment. The screen center was indicated by the red straight lines.

Supplementary note 12

Applicability of the process for assembling heterostructures based on CVD-grown 2D crystals.

We comment on the applicability of this procedure for assembling heterostructures based CVD-grown 2D crystals. This system is in principle applicable to the assembly of heterostructures based on CVD grown 2D crystals. Indeed, we have succeeded in picking up the CVD-grown 2D crystals by using the same stamping procedure in the ambient condition [Y. Hoshi *et al*, PRB 95, 241403 (2017)].
