# Supplementary material for: Autonomous robotic searching and assembly of two-dimensional crystals to build van der Waals superlattices
Source: Nat Commun. 2018 Apr 12;9:1413. doi: 10.1038/s41467-018-03723-w (PMC5897399; doi:10.1038/s41467-018-03723-w)
Supplement: Supplementary file 3 — Description of Additional Supplementary Files(PDF 168 kb) [file 41467_2018_3723_MOESM3_ESM.pdf]

Description of Additional Supplementary Files

File Name: Supplementary Movie 1
Description: Supplementary Movie of 2DMMS-Finder in operation (overall picture).

File Name: Supplementary Movie 2
Description: Supplementary Movie of 2DMMS-Finder in operation (screenshots).

File Name: Supplementary Movie 3
Description: Supplementary Movie of transferring chip in 2DMMS-Stamper.

File Name: Supplementary Movie 4
Description: Supplementary Movie of 2DMMS-Stampler (overall picture).

File Name: Supplementary Movie 5
Description: Supplementary movie of alignment procedure in 2DMMS-Stamper (screenshots).
